# Loss-of-Function Models of the Metabotropic Glutamate Receptor Genes *Grm8a* and *Grm8b* Display Distinct Behavioral Phenotypes in Zebrafish Larvae (*Danio rerio*)

**DOI:** 10.3389/fnmol.2022.901309

**Published:** 2022-06-13

**Authors:** Teresa M. Lüffe, Moritz Bauer, Zoi Gioga, Duru Özbay, Marcel Romanos, Christina Lillesaar, Carsten Drepper

**Affiliations:** Child and Adolescent Psychiatry, Center of Mental Health, University Hospital of Würzburg, Würzburg, Germany

**Keywords:** nervous system, brain disorders, psychiatric disorders, brain development, excitatory/inhibitory imbalance, metabotropic glutamate (mGlu) receptor

## Abstract

Members of the family of metabotropic glutamate receptors are involved in the pathomechanism of several disorders of the nervous system. Besides the well-investigated function of dysfunctional glutamate receptor signaling in neurodegenerative diseases, neurodevelopmental disorders (NDD), like autism spectrum disorders (ASD) and attention-deficit and hyperactivity disorder (ADHD) might also be partly caused by disturbed glutamate signaling during development. However, the underlying mechanism of the type III metabotropic glutamate receptor 8 (mGluR8 or GRM8) involvement in neurodevelopment and disease mechanism is largely unknown. Here we show that the expression pattern of the two orthologs of human *GRM8*, *grm8a* and *grm8b*, have evolved partially distinct expression patterns in the brain of zebrafish (*Danio rerio*), especially at adult stages, suggesting sub-functionalization of these two genes during evolution. Using double *in situ* hybridization staining in the developing brain we demonstrate that *grm8a* is expressed in a subset of *gad1a*-positive cells, pointing towards glutamatergic modulation of GABAergic signaling. Building on this result we generated loss-of-function models of both genes using CRISPR/Cas9. Both mutant lines are viable and display no obvious gross morphological phenotypes making them suitable for further analysis. Initial behavioral characterization revealed distinct phenotypes in larvae. Whereas *grm8a* mutant animals display reduced swimming velocity, *grm8b* mutant animals show increased thigmotaxis behavior, suggesting an anxiety-like phenotype. We anticipate that our two novel metabotropic glutamate receptor 8 zebrafish models may contribute to a deeper understanding of its function in normal development and its role in the pathomechanism of disorders of the central nervous system.

## Introduction

The main excitatory neurotransmitter in the vertebrate nervous system is L-glutamate, which mediates its function through binding to ionotropic (AMPA, NMDA, and kainate receptors), and metabotropic receptors. Whereas ionotropic receptors are responsible for the fast action of glutamate, metabotropic receptors have slower and longer-lasting effects on neurotransmission. The latter group of eight subtypes of metabotropic glutamate receptors (mGlu receptors or GRMs) identified in mammalian species so far can be subdivided into three distinct classes due to pharmacological properties, sequence homology, cellular localization, and G protein coupling (Niswender and Conn, 2010). The family type I receptors (mGluR1 and mGluR5) are mainly found at postsynaptic sites where they couple to Gα_q_ and Gα_11_ in order to activate downstream phospholipase Cβ1 and release intracellular Ca^2+^ and activate protein kinase C (PKC). In contrast, type II (mGluR2 and mGluR3) and type III (mGluR4, mGluR6, mGluR7 and mGluR8) receptors are coupled to Gα_i/o_ and act mainly on the adenylyl cyclase to control cAMP levels. While mGluR type II and mGluR type III receptors are associated with similar downstream signaling pathways, they differ substantially in their synaptic localization. Type III mGluRs (except for mGluR6) are predominantly expressed at the presynapse, thereby regulating excitatory (glutamatergic), inhibitory (GABAergic) and neuromodulatory transmitter release. In contrast, type II mGluRs are located outside of the release zone of the pre- and postsynapse (Niswender and Conn, 2010; Bodzęta et al., 2021).

All mGluRs contain a large extracellular domain that comprises the agonist-binding Venus fly trap (VFT) domain and a cysteine-rich domain that connects to the highly conserved seven-pass trans-membrane domain (Pin and Bettler, 2016). mGluRs are constitutive dimers which are linked via disulfide bonds in the proximity of the VFT (Niswender and Conn, 2010). The complexity of mGluRs function and mode of action in the nervous system is enhanced by alternative splicing to generate multiple isoforms of single mGluRs, homo- and heterodimerization of different mGluRs to change its signaling properties, cell type-specific protein interactions and varying downstream signaling cascades (Niswender and Conn, 2010; Pin and Bettler, 2016; Bodzęta et al., 2021; McCullock and Kammermeier, 2021).

Over the past years, evidence indicates that mGluRs are involved in several disorders of the nervous system. In neurodegenerative disorders like Parkinson’s disease, Alzheimer’s disease and Huntington’s disease involvement of mGluRs, including the type III receptor GRM8, were documented frequently (Ribeiro et al., 2017). In neurodevelopmental disorders, an association of copy number variation of the *GRM8* locus with ADHD was found (Elia et al., 2012; Akutagava-Martins et al., 2014; Hawi et al., 2015; Liu et al., 2021). A 1.9 Mb microdeletion on chromosome 7 q including the *GRM8* locus was associated with intellectual disability and signs of autism (Sangu et al., 2017). Using GWAS, an association of *GRM8* genotype and trait depression was detected (Terracciano et al., 2010). In a Han Chinese population, a link with major depression and schizophrenia was demonstrated for SNPs located in the *GRM8* gene locus (Li et al., 2016). A similar result was found in an Iranian population for *GRM8* and schizophrenia (Tavakkoly-Bazzaz et al., 2018). The association of the *GRM8* locus and major depression was later confirmed in a massive meta-analysis of several GWAS studies (Howard et al., 2019). In substance use conditions, associations with *GRM8* were shown as well (Long et al., 2015; Bauer and Covault, 2020).

Several animal models have been developed to elucidate mGluR8 function. The very first study on mGluR8 knockout mice revealed an anxiolytic-like phenotype in a conditioned fear model (Gerlai et al., 2002). Further studies have either demonstrated an anxiogenic-like behavior (Linden et al., 2002, 2003; Robbins et al., 2007; Duvoisin et al., 2010a,b) or no increase in anxiety-like behavior (Fendt et al., 2010; Davis et al., 2013), probably being an age, developmental stage or background strain dependent effect in the different models generated. For instance, in older mGluR8 knockout mice (6 month of age) it was shown that increased measures of anxiety were present (Linden et al., 2002; Duvoisin et al., 2005), whereas in younger mice (2–4 months of age) no differences were found in the same test (Raber and Duvoisin, 2015). Another behavioral dimension often characterized to investigate psychiatric conditions like ADHD are activity-related phenotypes. The mGluR8 knock-out mice are either being hypoactive (Duvoisin et al., 2005), hyperactive (Gerlai et al., 2002) or have not shown any alteration of motor behavior (Linden et al., 2002; Robbins et al., 2007; Raber and Duvoisin, 2015). Besides genetic models, pharmacological models have been developed to investigate mGluR8 function, demonstrating alterations in terms of alcohol dependence and anxiety-like phenotype (Bahi, 2017). Therefore, the currently available animal models do not draw a consistent picture of mGluR8 function in psychiatric relevant behavioral modulation.

Zebrafish (*Danio rerio*), an increasingly accepted vertebrate model organism, is widely used in for instance toxicology, pharmacology, neuroscience and developmental research. The conserved vertebrate brain organization and neurochemistry, the rich behavioral repertoire, the high number of offspring, the comparably fast development, the short generation times and the genetic homology to human makes zebrafish an attractive model organism (Stewart et al., 2014, 2015). Especially, screening for new compounds for pharmacological interventions on larger scales is a huge benefit over other vertebrate model organisms (Rennekamp and Peterson, 2015).

The aim of this study is to explore the consequences of mGluR8 deficiency to understand its role in neurodevelopment and potential contribution to behaviors relevant for neurodevelopmental psychiatric disorders using zebrafish larvae. In the zebrafish genome there are two mGluR8 paralogs encoded by *grm8a* and *grm8b*. We characterize the expression pattern of *grm8a* and *grm8b* at early and late developmental stages until adulthood with a particular focus on the nervous system to understand spatial and temporal differences. This information will be valuable to evaluate behavioral differences and gain insight into neuronal dysfunction of both proteins. Next, we generate loss-of-function mutants of the two paralogs using CRISPR/Cas9. The mutants are viable without any gross morphological abnormalities which makes them suitable for further behavioral characterizations. An initial basic behavioral assessment at larval stage concludes our analysis where we found first hints that mGluR8-deficiency can cause psychiatric relevant phenotypes in zebrafish.

## Experimental Procedures

### Animal Handling

All experiments were performed in the zebrafish (*Danio rerio*) AB/AB wildtype strain background (zfin id.: ZDB-GENO-960809-7). Monoaminergic cells expressing *grm8a* were identified in the enhancer trap line *Tg(Etvmat2:GFP)* (Wen et al., 2008). Larvae were raised at 28°C with a light/dark cycle of 14/10 h in Danieau’s solution with or without methylene blue (Cold Spring Harb. Protoc, 2011)^1^. To suppress pigmentation for whole-mount RNA *in situ* hybridization (ISH) and immunohistochemistry, Danieau’s solution containing 0.2 mM 1-phenyl-2-thiourea was used. Embryo staging was performed according to Kimmel et al. (1995). Embryos were manually dechorionated and fixed in 4% paraformaldehyde (PFA) in 1x phosphate-buffered saline (PBS). For adult brain preparations, fish were euthanized with an overdose of MS-222 and decapitated. The heads were fixed over night at 4°C in 4% PFA before the brains were dissected and post-fixed for another 4 h at room temperature (RT). All animal handling was performed in accordance with the regulations for animal welfare of the District Government of Lower Franconia, Germany.

### Whole-Mount RNA *in situ* Hybridization

A *grm8a* target sequence was reverse transcription-PCR amplified with RevertAid reverse transcriptase (Thermo Fisher Scientific, Waltham, MA, United States) and an oligodT primer (for gene-specific primer sequences see Supplementary Table 1), cloned into pCRII-TOPO vector (Thermo Fisher Scientific, Waltham, MA, United States) and verified by Sanger sequencing. Cloned full-length *grm8b* cDNA was a kind gift from Marion Haug and Stephan Neuhauss (Haug et al., 2013). The resulting plasmids were linearized (*Not*I, Thermo Fisher Scientific, Waltham, MA, United States) and purified (GenElute PCR Clean-Up Kit, Merck KGaA, Darmstadt, Germany) before *in vitro* transcribed with SP6 RNA polymerase and digoxiginin (DIG) RNA Labeling Mix (Merck KGaA, Darmstadt, Germany). The final RNA probes were purified by LiCl and ethanol precipitation. Before usage, the *grm8b* probe was hydrolyzed for 20 min at 60°C and 30 min in –20°C and precipitated by LiCl and ethanol again.

Whole-mount RNA ISH was performed according to the methods published previously (Thisse and Thisse, 2008; Lechermeier et al., 2019, 2020; Lüffe et al., 2021). Briefly, specimens were fixed in 4% PFA, washed in PBS containing 0.1% Tween-20 (PBST) and dehydrated in methanol (MeOH) and finally stored in 100% MeOH at –20°C. For ISH, the samples were rehydrated and then permeabilized by Proteinase K treatment (10 μg/ml). The specimens were then post-fixed in 4% PFA, washed in PBST and were incubated in hybridization buffer containing 5 mg/ml torula yeast RNA type VI (Merck KGaA, Darmstadt, Germany) for 1 h at 65°C. Subsequently, the samples were incubated in hybridization buffer containing the RNA ISH probe (dilution 1:100) overnight at 65°C. On the next day, the samples were passed through stringency washes at 65°C in hybridization buffer with saline-sodium citrate (SSC) buffer with a last step in 0.05 × SSC (in PBST) for 1 h at 65°C. For subsequent immuno detection of DIG, the samples were incubated in ISH blocking buffer [PBST with 2% normal sheep serum (NSS) and 2 mg/ml bovine serum albumin (BSA)] and then for 2 h at RT in sheep anti-DIG Fab fragments conjugated with Alkaline Phosphatase (AP; anti-DIG-AP, Merck KGaA, Darmstadt, Germany) diluted 1:5,000. After extensive washes in PBST, the samples were rinsed in alkaline tris-buffer (pH 9.5) for 30 min at RT. The AP activity was visualized with nitroblue tetrazolium/5-bromo-4-chloro-3-indolylphosphate (NBT/BCIP) (Merck KGaA, Darmstadt, Germany). Finally, the enzymatic reaction was stopped, and samples were transferred into 80% glycerol in PBST for long-term storage in darkness.

Dissected adult brains were processed as described above. Before incubating in ISH blocking buffer, the brains were embedded in 3% agarose in PBS and cut into transverse sections of 80 μm using a vibratome (Vibratome Series 1,000 Sectioning System). Subsequently, the brain slices were processed as described for whole-mount RNA ISH above.

For two-color RNA ISH, a *gad1a* (previously *gad67a*) containing plasmid (Martin et al., 1998) was linearized (*Eco*RI, Thermo Fisher Scientific, Waltham, MA, United States), purified (GenElute PCR Clean-Up Kit, Merck KGaA, Darmstadt, Germany) and *in vitro* transcribed using T3 RNA polymerase and fluorescein (FLUO) RNA Labeling Mix (Merck KGaA, Darmstadt, Germany). DIG- and FLUO-labeled probes were diluted together (1:100 each) in hybridization buffer. After hybridization, stringency washes and blocking, the samples were incubated in sheep anti-FLUO-AP Fab fragments diluted 1:2,000 in ISH blocking buffer. Subsequent to several washes in tris-buffer (pH 8.2) the AP activity was visualized with SIGMAFAST fast red TR/naphthol AS-MX phosphate tablets (4-chloro-2-methylbenzenediazonium/3-hydroxy-2-naphthoic acid 2,4-dimethylanilide phosphate) in 0.1 M Trizma buffer (Merck KGaA, Darmstadt, Germany). The reaction was stopped in PBST, and the anti-FLUO-AP Fab fragments were detached in PBST at 68°C for 2 h. Afterward, blocking, anti-DIG-AP immunolabeling and visualization of AP activity was performed as described before.

### Cryosections

Samples were washed and cryoprotected in PBS containing 15% sucrose overnight at 4°C. Embedding was done in 7.5% gelatine dissolved in 15% sucrose in PBS. Blocks were cut and snap frozen in 2-methylbutane pre-cooled with liquid nitrogen. On a cryostat (Microm HM 500 OM), cryoblocks were cut transversally with a thickness of 20 μm and sections were mounted on slides and cover slipped with 80% glycerol. The samples were stored in darkness at 4°C until image acquisition.

### Generation and Genotyping of *Grm8a* and *Grm8b* Mutant Lines

The selection of the CRISPR/Cas9 target sites were done with CHOPCHOP (Labun et al., 2019). Oligonucleotides for the synthesis of the single guide RNA (sgRNA) (Supplementary Table 1) were annealed and ligated with the linearized (*Eco*31I/*Bsa*I, Thermo Fisher Scientific, Waltham, MA, United States) pDR274 vector (Addgene #42250). Sequences were verified with Sanger sequencing (Eurofins Genomics, Ebersberg, Germany). The plasmid containing the sgRNA sequence was linearized (*Dra*I, Thermo Fisher Scientific, Waltham, MA, United States) and *in vitro* transcribed with a custom-made T7 RNA polymerase (a kind gift from Thomas Ziegenhals and Utz Fischer). The sgRNA was purified by Roti-phenol/chloroform/isoamylalcohol (Carl Roth GmbH & Co., KG, Karlsruhe, Germany) and injected (100 ng/μl) together with the Cas9-NLS protein (300 ng/μl, *S. pyogenes*, New England Biolabs, Ipswich, MA, United States) into the animal pole of fertilized one-cell stage zebrafish eggs. Successful targeting events were confirmed by PCR on extracted gDNA (primer sequences in Supplementary Table 1) and subsequent Sanger sequencing (Eurofins Genomics, Ebersberg, Germany). Germline transmission of indel mutations were verified by outcrossing with AB/AB wildtypes and subsequent genotyping of F_1_ larvae. Positive F_0_ fish were used to obtain the F_1_ generation by additional outcrossing. Individual *grm8a* and *grm8b* F_1_ adult mutants were used to analyze the induced mutations and establish the lines. Finally, the lines containing the deletion mutations were selected. For final mutation verification, gDNA of *grm8a^–/–^* and *grm8b^–/–^* F_3_ mutants were PCR amplified with primers flanking the sgRNA target site and Sanger sequenced. For the experiments described below, F_3_ embryos and larvae were used.

### Immunohistochemistry

Whole-mount immunohistochemical stainings were performed on embryos at 24 hpf with the yolk removed. The genotype of immuno-stained *grm8a* and *grm8b* mutants was determined using the tail of each embryo for gDNA extraction and subsequent genotyping as described above.

#### Anti-cleaved Caspase 3 (cCasp3)

After fixation in 4% PFA for 3 h at RT embryos were dehydrated through a MeOH series [in PBS with 0.8% Triton X-100 (0.8% PBST)] and finally stored in 100% MeOH. Following permeabilization in 100% acetone for 7 min at –20°C, the samples were rehydrated in 50% MeOH in 0.8% PBT for 1 h at –20°C and subsequently washed in ddH_2_O and 0.8% PBST at RT. Blocking was done for 1 h at RT in blocking buffer (0.8% PBST with 1% DMSO, 10% NSS and 2 mg/ml BSA), before the samples were labeled for 3 days at 4°C with a polyclonal rabbit anti-cleaved Caspase-3 (Asp175) primary antibody (#9661, Cell Signaling Technology, Danvers, MA, United States; RRID:AB_2341188) diluted 1:500 in blocking buffer. After several washing steps in 0.8% PBT, the samples were exposed to secondary goat anti-rabbit IgG (H + L) conjugated to Alexa Fluor 488 (A-11034, Thermo Fisher Scientific, Waltham, MA, United States; RRID:AB_2576217) diluted 1:1,000 in blocking buffer for 2 days at 4°C. Finally, after extensive washing in 0.8% PBT the samples were stored in 80% glycerol (in PBST) in darkness at 4°C.

### Quantitative Real-Time PCR

Quantitative real-time RT-PCR (qPCR) was performed on 5 dpf old ^–/–^, ^+/–^ and ^+/+^ siblings of *grm8a* and *grm8b* mutant lines. Genomic DNA extraction was done on tail cuts for subsequent genotyping of individual embryos. The whole body including the head was used for total RNA extraction and subsequent cDNA synthesis using standard methods. RNA from 10 pooled embryos were isolated for each genotype. For the qPCR analysis each target gene is represented by three technical replicates of each biological sample (*n* = 3) for each genotype. Additionally, a no RT control (NRT) and no template control (NTC) served as negative controls. Quantification and calculation were done with the comparative Ct (2^–ΔΔCt^) method using *actb1* and *gapdh* as housekeeping genes (Supplementary Table 1). Group differences were determined by applying a one-way ANOVA with a significance level of 0.05.

### Image Acquisition and Processing

A Zeiss Axiophot light microscope equipped with a Zeiss AxioCam MRc digital camera and the AxioVision Rel.4.8 Ink. software was used for image acquisition of RNA ISH labeling on embryonic and larval whole-mounts as well as on adult brain sections. For adult brain sections, single images were merged using a grid/collection ImageJ plugin (Preibisch et al., 2009). Size measurements were done on images taken at a Leica M205 FA fluorescence microscope equipped with a Leica DFC 420C digital camera and the Leica Application Suite V3.8 Ink. software. Images were recorded through a Leica Planapo 1.0x objective (Leica Camera AG, Wetzlar, Germany). Size measurements were done on fixed embryos (24 hpf) using the ImageJ image processing package Fiji 2.0.0 (Schindelin et al., 2012). Head area was traced using the midbrain-hindbrain boundary as posterior boundary. Yolk diameter was defined as the distance between most ventral center of the eye and the edge between circular and elongated yolk sac. Length measurements were calculated between the dorsal part of the midbrain-hindbrain boundary and the most posterior end of the tail not considering the fin. Size parameters are all given in squared pixels (pixel^2^). Image acquisition of anti-cCasp3 stainings was performed at a Zeiss LSM 780 confocal microscope equipped with a Lasos Argon 488 nm laser and the ZEN 2012 SP1 software. Images were taken through a Plan APO 20x/0.8 objective. Brightness adjustments of confocal images were done in Fiji 2.0.0. Images were arranged into final figures by using the vector graphics software Inkscape 1.0.1.

### Locomotor Tracking

Locomotion tracking was performed on 5 dpf old larvae using the semi-automatic system ZebraBox and the corresponding commercial software ZebraLab (ViewPoint, Lyon, France). For the *grm8a* mutant line larvae from 6 clutches were analyzed on 6 different days. For the grm8b mutant line larvae from 10 clutches were used on 10 different days. Individual larvae were placed in 12-well plates containing 1 ml of Danieau’s solution. Temperature was kept constant at 28°C and swimming tracks were recorded in the dark (infrared illumination of 850 nm) with an infrared camera with 30 fps (frames per second). Three activity levels were defined with the following thresholds: inactive, < 0.2 cm/s; low activity, 0.2 cm/s < and < 1 cm/s; high activity, > 1 cm/s. Larvae were tracked for a total duration of 10 min, separated into a 5 min habituation and a 5 min test phase. Only data collected during the test phase was considered for final group comparisons. Locomotor activity was determined based on four different parameters: Total distance swum, mean velocity during low/high activity or both (total mean velocity), duration of inactivity, low or high activity and the number of events during inactive, low, and high activity phase.

To analyze thigmotaxis behavior, data obtained during locomotor assays were replayed using the commercial tracking software. Therefore, each region of interest/well (ROI) was virtually divided into two different zones: An outer zone with a width of 4 mm and an inner zone with a radius of 7.35 mm. An increased percentage of time spent in the outer zone of the ROI was defined as increased thigmotaxis behavior.

### Data Analysis and Statistics

RStudio (RStudio 1.3.959, at https://rstudio.com/) was used for data analysis. Normal distribution was checked with the Shapiro-Wilk’s test. According to distribution, group differences were calculated by one-way ANOVA or Kruskal-Wallis rank sum test followed by Tukey’s HSD *post-hoc* test for parametric and Dunn’s *post hoc* test for non-parametric data. The Benjamini-Hochberg adjustment was used to control the false discovery rate (FDR). The general significance level was set to 0.05. Z-score transformation was used to compare data obtained with two different versions of the tracking software ZebraLab. Effect sizes were calculated by the Cliff’s delta (Supplementary Table 4) using the *effsize* package in RStudio (Torchiano, 2016). Appropriate sample sizes were determined by using the software G*Power 3.1.9.4 (Faul et al., 2007, 2009) with α and β set to 0.05.

## Results

### Two Orthologs of the Human *GRM8* Gene Are Present in the Zebrafish Genome

Due to a probable genome duplication in the teleost lineage during evolution (Meyer and Schartl, 1999; Glasauer and Neuhauss, 2014) two orthologs of the human *GRM8* gene (ENSG00000179603) are present in the zebrafish genome (Haug et al., 2013). The human gene is located on chromosome 7 and consists of 11 exons, encoding a protein of 908 aa and a molecular mass of 101.7 kDa. The two zebrafish orthologs are located on chromosome 4 (*grm8a*, ENSDARG00000077654) and chromosome 25 (*grm8b*, ENSDARG00000076508), respectively. *grm8a* is a 10 exon gene producing a protein of 909 aa with a molecular mass of 101.7 kDa. *grm8b* consists of 11 exons and encodes a protein of 907 aa with a molecular mass of 100.9 kDa. Both zebrafish protein sequences are matching the human sequence to 81.08% for Grm8a and 80.26% for Grm8b. The overall protein structure of zebrafish Grm8a and Grm8b are similar and share 86.55% sequence identity, whereas especially in the N-terminal part of the two proteins the biggest sequence variation can be detected (data not shown). Interestingly, using protein sequences from multiple species we detected a loss of an evolutionary conserved tyrosine residue in the C-terminal domain of Grm8b, despite an overall similar structural conservation of both zebrafish proteins across species (data not shown).

### Early Developmental Expression of *Grm8a* and *Grm8b*

**FIGURE 1:**
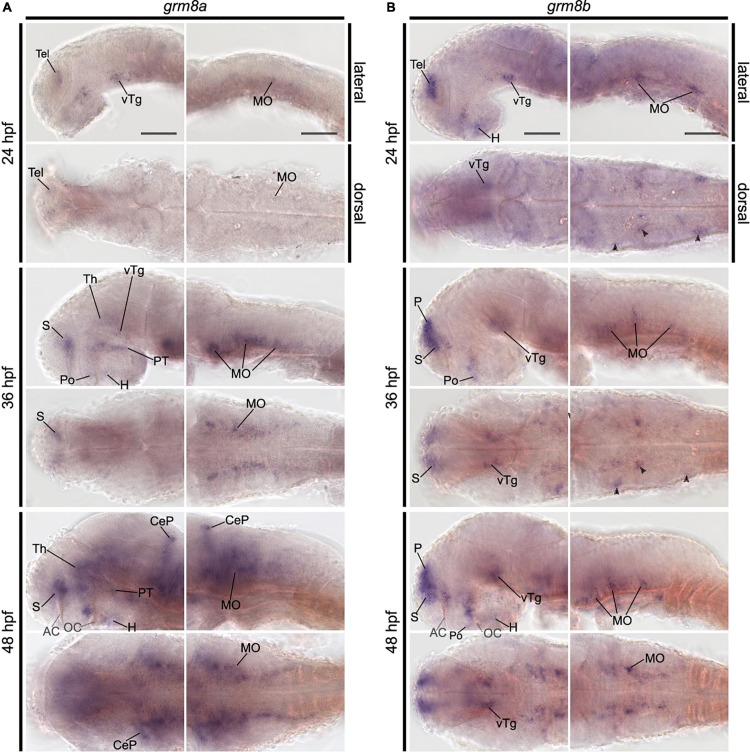
Pattern of *grm8a* and *grm8b* expression in the developing zebrafish revealed by whole-mount RNA *in situ* hybridization. **(A)** *Grm8a* expression at 24, 36, and 48 hpf in the developing zebrafish. Lateral (top) and dorsal (bottom) overviews indicate expression as early as 24 hpf in the central nervous system. **(B)** *Grm8b* expression at 24, 36, and 48 hpf in the developing zebrafish. Lateral (top) and dorsal (bottom) overviews indicate prominent expression as early as 24 hpf in the central nervous system. Details on the individual expression patterns are described in the main text. Arrowheads indicate transcript labeling in individual cell cluster of the medulla oblongata (MO). All images are oriented with anterior to the left. Abbreviations are listed in Table 1. Scale bar is 100 μm.

Expression of *grm8a* and *grm8b* were shown in the CNS of 3 dpf and 5 dpf old zebrafish before (Haug et al., 2013). However, a detailed description of the developmental trajectory including adult stages is missing. This information is crucial to understand the potential impact of null mutations of both genes on neurodevelopment and behavior. Therefore, we used RNA *in situ* hybridization (ISH) to characterize the expression patterns of both genes. We focused our analysis first on early developmental stages and found restricted expression at early and broad expression at advanced developmental stages for both paralogs, although the temporal differences are more pronounced for *grm8a* compared to *grm8b*. Transcripts for both paralogs were detected first at 24 hpf in the telencephalon and ventral tegmentum, and for *grm8b* in the hypothalamus and the medulla oblongata (Figures 1A,B and Supplementary Figures 1A–S, 2A–S). Similar or partly overlapping expression patterns for *grm8a* and *grm8b* were observed in the telencephalon (30–72 hpf), the ventral tegmentum (24–72 hpf), the optic tectum (72 hpf), and the ganglion and inner nuclear layer of the retina (72 hpf) whereas expression in the preoptic region (*grm8a*: 36–72 hpf; *grm8b*: 30–72 hpf) and the hypothalamus (*grm8a*: 30–72 hpf; *grm8b*: 24–72 hpf) shows temporal discrepancies potentially attributable to staining quality.

Further brain regions with prominent temporal discrepancies between *grm8a* and *grm8b* expression comprise the thalamus and the olfactory bulb. *grm8a* expression in thalamic regions is detected at 36 hpf (Figure 1A) in contrast to first *grm8b* transcript labeling at 72 hpf (Supplementary Figures 2I,O). Further, until 72 hpf distinct *grm8b* transcript labeling is detected in the most anterior telencephalon (comprising the developing olfactory bulbs) (Supplementary Figures 2B,D,F,H,J), whereas *grm8a* expression is absent or only detected faintly for earlier stages than 72 hpf (Supplementary Figures 1B,D,F,H,J).

Differences in both temporal and spatial distribution were observed for expression in the medulla oblongata. While *grm8a* transcripts were labeled in a bilateral stripe-like pattern from 30 hpf (Figure 1A and Supplementary Figures 1C,D,K–N), *grm8b* transcript labeling is restricted to scattered cell clusters at 24, 30, and 36 hpf (Figure 1B and Supplementary Figures 2A–F,K–N). At 48 hpf, *grm8b* expression also adapts a stripe-like pattern (Figure 1B and Supplementary Figures 2G,H). Differences in the spatial distribution comprises *grm8a* expression in the medial anterior medulla oblongata at 72 hpf (Supplementary Figure 1R), a subregion suggested to be implicated in the integration of sensory and modulatory input and in providing regulatory output onto (pre-) motor areas (Naumann et al., 2016).

Although many brain regions show similar expression patterns or only minor temporal or spatial differences in the localization of the two transcripts, distinct expression of *grm8a* or *grm8b* is also detected for some areas in the central nervous system. For instance, the posterior tuberculum is exclusively labeled for *grm8a* with expression starting as early as 30 hpf (Supplementary Figure 1K), the cerebellum with first detection at 48 hpf (Figure 1A), and the pretectum with transcripts labeled at 72 hpf (Supplementary Figures 1O,Q). Notably, like previously mentioned for the medial anterior MO, all identified regions with distinct *grm8a* expression were demonstrated to be involved in sensorimotor integration and/or motor control in the past (Ahrens et al., 2012; Naumann et al., 2016; Jha and Thirumalai, 2020). In the pallium distinct *grm8b* expression was observed (Figure 1B and Supplementary Figure 2K). Interestingly, this area is suggested to comprise homologous structures to the tetrapod cortex, the amygdala, and the hippocampus (Mueller et al., 2011; Ganz et al., 2014).

Taken together, the presented findings confirm *grm8a* and *grm8b* expression in the CNS of developing zebrafish (Supplementary Table 2). Besides, temporal, and spatial discrepancies in the expression patterns suggest that *grm8a* and *grm8b* may take over common and distinct functional roles during (neuro-) development.

**TABLE 1:** List of abbreviations of anatomical terms.

Abbreviations	Anatomical structure
AC	Anterior commissure
CC	Cerebellar crest
CCe	Cerebellar corpus
CeP	Cerebellar plate
Cpost	Posterior commissure
DIL	Diffuse nucleus of the inferior lobe
DON	Descending octaval nucleus
DT	Dorsal thalamus
DTN	Dorsal tegmental nucleus
EG	Granular eminence
EN	Entopeduncular nucleus
GCL	Ganglion cell layer of retina
H	Hypothalamus
Ha	Habenula
Hc	Caudal zone of periventricular hypothalamus
Hd	Dorsal zone of periventricular hypothalamus
Hv	Ventral zone of periventricular hypothalamus
INL	Inner nuclear layer
LCa	Caudal lobe of cerebellum
LH	Lateral hypothalamic nucleus
LLF	Lateral longitudinal fascicle
LVII	Facial lobe
LIX	Glossopharyngeal lobe
LX	Vagal lobe
MLF	Medial longitudinal fascicle
MO	Medulla oblongata
MON	Medial octavolateralis nucleus
NIII	Oculomotor nucleus
NIV	Trochlear nucleus
NLV	Nucleus lateralis valvulae
NXm	Vagal motor nucleus
OB	Olfactory bulb
OC	Otic capsule
ORR	Optic recess region
OT	Optic tract
P	Pallium
PG	Preglomerular area
PGZ	Periventricular gray zone of optic tectum
Po	Preoptic region
PP	Periventricular pretectal nucleus
Pr	Pretectum
PT	Posterior tuberculum
PTN	Posterior tuberal nucleus
PVO	Paraventricular organ
Ra	Raphe
RF	Reticular formation
RV	Rhombencephalic ventricle
S	Subpallium
SC	Spinal cord
Tel	Telencephalon
TeO	Optic tectum
TeV	Tectal ventricle
Tg	Tegmentum
Th	Thalamus
TL	Longitudinal torus
TLa	Lateral torus
TPp	Periventricular nucleus of posterior tuberculum
TTB	Tractus tectobulbaris
TS	Semicircular torus
Va	Valvular cerebelli
VIII	Octaval nerve
VT	Ventral thalamus
vTg	Ventral tegmentum
X	Vagal nerve
Y	Yolk

### Adult Expression of *Grm8a* and *Grm8b* in the Zebrafish Brain

**FIGURE 2:**
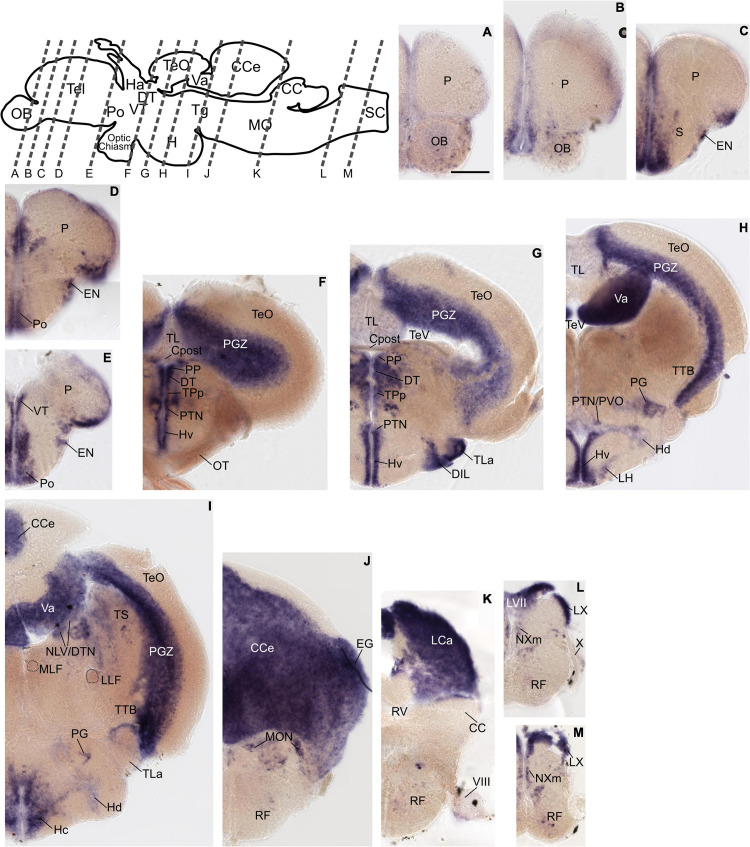
*Grm8a* expression pattern in adult zebrafish brain labeled by RNA *in situ* hybridization. **(A–M)** *Grm8a* transcript labeling by RNA *in situ* hybridization on cross-sections of an adult zebrafish brain. Images are arranged from anterior to posterior as indicated by the scheme at the top. Details on *grm8a* expression pattern are described in the main text. Anatomical abbreviations are listed in Table 1. Scale bar is 200 μm.

**FIGURE 3:**
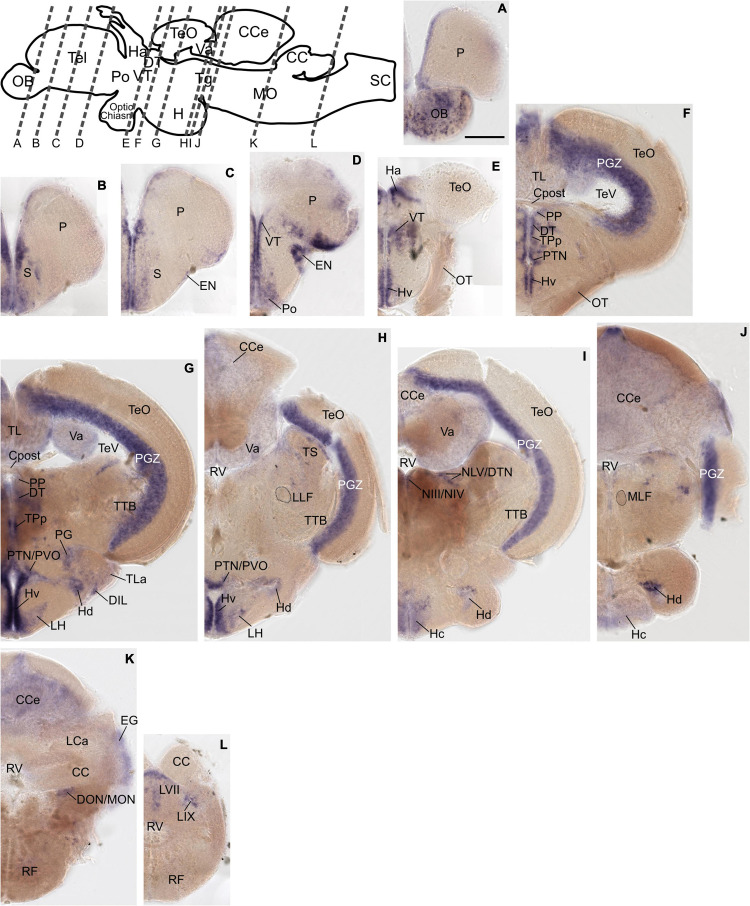
*Grm8b* expression pattern in adult zebrafish brain labeled by RNA *in situ* hybridization. **(A–L)** *Grm8b* transcript labeling by RNA *in situ* hybridization on cross-sections of an adult zebrafish brain. Images are arranged from anterior to posterior as indicated by the scheme at the top. Details on *grm8b* expression pattern are described in the main text. Anatomical abbreviations are listed in Table 1. Scale bar is 200 μm.

The previously distinct expression pattern for *grm8a* or *grm8b* become increasingly similar with further development into adulthood. This can be best illustrated in the pallium, the thalamus, and the posterior tuberculum. Distinct expression domains, observed during early embryonic development (Figures 1A,B and Supplementary Figures 1A–H, 2A–H) align partially by 72 hpf (Supplementary Figures 1I,J, 2I,J) and fully when reaching adulthood (Figures 2, 3). At adult stage, *grm8a* and *grm8b* transcripts are both detected along the ventricular side of the pallium (P, Figures 2A–E, 3A–D), in the ventral (VT, Figures 2E, 3D,E) and dorsal thalamus (DT, Figures 2F,G, 3F,G) and the periventricular nucleus of the posterior tuberculum (TPp, Figures 2F,G, 3F,G) and the area comprising the posterior tubercular nucleus or the paraventricular organ (PTN/PVO, Figures 2F–H, 3F–H).

Expression domains with similar expression of *grm8a* and *grm8b* in the early developmental phase maintain the expression similarity of both paralogs till adulthood. This applies for instance to expression in the subpallium (S) revealed in the central, ventral, dorsal, and entopeduncular nuclei (EN, Figures 2C–E, 3B–D), the preoptic region (Po, Figures 2D,E, 3D), the hypothalamus with expression in the diffuse nucleus of the inferior lobe (DIL, Figures 2G, 3G), the lateral hypothalamic nucleus (LH, Figures 2H, 3H) and the ventral, dorsal and caudal zone of the periventricular hypothalamus (Hv, Hd, Hc, Figures 2F–I, 3E–J), the tegmentum [with expression in the area encompassing the lateral valvulae and dorsal tegmental nuclei (NLV/DTN, Figures 2I, 3I)] and the optic tectum [with expression in the periventricular gray zone (PGZ, Figures 2F–I, 3F–J)].

Not only expression similarities are maintained into adulthood, discrepancies are too. For instance, the less pronounced *grm8b* expression detected in the developing cerebellum is still observed in the mature brain [with expression in the corpus cerebelli (CCe, Figures 2I–K, 3H–K), the valvular cerebelli (Va, Figures 2H,I, 3G–I), the medial octavolateralis nucleus (MON, Figures 2J, 3K) and the eminentia granularis (EG, Figures 2J, 3K)]. Similarly, the weak *grm8b* expression is detectable in the developing and mature pretectum [periventricular pretectal nucleus (PP, Figures 2F,G, 3F,G)]. Notably, other regions in the mature brain with exclusive expression of *grm8a* such as the reticular formation (RF), the vagal lobe (LX), and the torus semicularis (TS) (Figures 2I–M) were demonstrated to be connected to the pretectum and/or cerebellum (Kaslin and Brand, 2016; Kramer et al., 2019). The restricted distribution of *grm8b* transcripts observed in the developing MO is maintained into adulthood as well. While the reticular formation (RF, Figures 2J–M), the vagal motor nucleus (NXm, Figures 2L,M) and the vagal (LX, Figures 2L,M) and facial lobe (LVII, Figure 2L) all express *grm8a*, expression of *grm8b* was only detected in the facial (LVII, Figure 2L) and the glossopharyngeal lobe (LIX, Figure 3L).

Other brain structures with comparably weak expression of *grm8b* include nuclei of the posterior tuberculum such as the torus lateralis (TLa, Figures 2G, 3G) and the preglomerular nuclei (PG, Figures 2H,I, 3G). While labeling of *grm8a* transcripts appear generally stronger and broader at 72 hpf (Supplementary Figures 1I,J,O–S) and in the adult brain (Figure 2), the opposite is true for transcript expression in the olfactory bulb throughout development (OB, Figure 3A).

### *Grm8a* Is Partly Expressed by a Subset of *Gad1a*-Positive Cells

Grm8, like other type III metabotropic glutamate receptors, is involved in the regulation of neurotransmitter release of various neuronal cell types. Therefore, it is crucial to determine the transmitter identity of Grm8-expressing cells in order to assess the effect of Grm8 loss-of-function on different transmitter systems.

**FIGURE 4:**
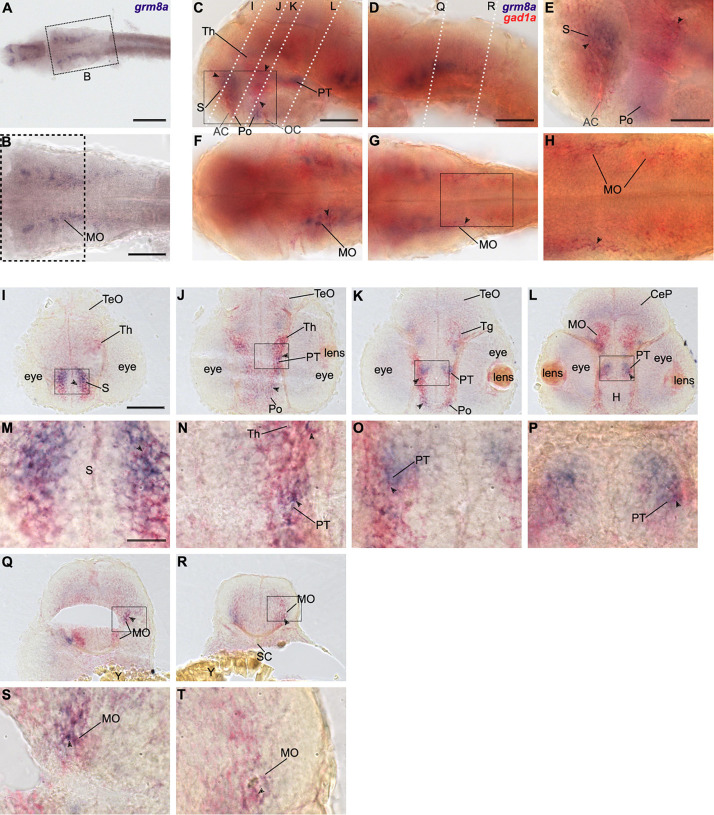
*Grm8a* is partly expressed in *gad1a* positive neurons in the developing zebrafish brain. **(A)** Whole-mount RNA *in situ* hybridization of *grm8a* expression at 36 hpf. **(B)** Magnified picture of the region boxed in **(A)**. Scale bar, 200 μm (overview) and 100 μm (magnification). **(C–T)** Double labeling of *grm8a* (blue) and *gad1a* (red) expression in 36 hpf old embryos using two-color RNA *in situ* hybridization. Lateral **(C,D)** and dorsal **(F,G)** overview of embryonic CNS with anterior to the left. **(E,H)** Magnifications of boxed areas in **(C,G)**. Dashed lines in **(C,D)** indicate cutting sites for cross sections shown in **(I–R)**. **(M–P,S,T)** Magnifications of boxed areas in **(I–L,Q,R)**, respectively. Arrowheads indicate sites of co-localization. Abbreviations are listed in Table 1. Scale bars: 100 μm (overview), 50 μm (magnified images).

Comparing to our previous findings we noted that the developmental distribution of *grm8a* resembles the one of *gad1a* and *gad1b* at 36 hpf (Lüffe et al., 2021), especially in the medulla oblongata (MO, Figures 4A,B). Hence, we hypothesized that *grm8a* expression might correlate with the distribution of GABAergic markers as seen for other species and brain regions before (Ferraguti et al., 2005). Therefore, we decided to investigate the distribution of *gad1a*, a well-known GABAergic marker in relation to *grm8a* expression. To confirm colocalization in single cells we used double RNA ISH for *gad1a* (red) and *grm8a* (blue) at 36 hpf on whole-mounts and cryosections. Due to the largely similar pattern of *gad1a* and *gad1b* expression (Lüffe et al., 2021), the technical benefits and the broader expression of *grm8a* compared to *grm8b* (Figures 1A,B, 2, 3), we decided to assess the colocalization of *grm8a* and *gad1a* expression.

Colocalization between *grm8a* and *gad1a* expression was detected throughout development in the subpallium (S, Figures 4C,E,I,M), the thalamus (Th, Figures 4C,I,J,N) and the medulla oblongata (MO, Figures 4F–H,L,Q–T). Further, the posterior tuberculum (PT, Figures 4C,J–L,N–P) and the preoptic region (Po, Figures 4C,E,J,K) showed an overlap of both expression patterns at 36 and 48 hpf. To further compare *grm8a* expression with other neuronal markers we used the *Tg(Etvmat2:GFP)* reporter line, which labels monoaminergic neurons (Wen et al., 2008). Interestingly, while *grm8a* and *gad1a* expression overlap extensively across several brain regions, the colocalization of *grm8a*-positive punctae and *vmat2*-expressing cells was restricted to only a few cells of the rostral/intermediate and caudal hypothalamus and the raphe nuclei (Supplementary Figure 3). Taken together, prominent colocalization of *grm8a* with *gad1a* was observed with high incidence in previously mentioned brain regions associated with motor functions, including the subpallium, the posterior tuberculum, the thalamus, the cerebellum, and the medulla oblongata.

### Generation of *Grm8a* and *Grm8b* Knock-Out Lines Using CRISPR/Cas9

Based on the expression of *grm8a* and *grm8b* transcripts in the brain of developing and mature zebrafish we hypothesized that both paralogs may have functional roles in the CNS of zebrafish throughout development. Until now, insights into Grm8 function exclusively originate from a few studies in mammals (Linden et al., 2002, 2003; Duvoisin et al., 2005; Schmid and Fendt, 2006; Woo et al., 2021) whereas functional data on Grm8 in zebrafish is missing. We therefore decided to generate both *grm8a* and *grm8b* knockout lines using CRISPR/Cas9.

**FIGURE 5:**
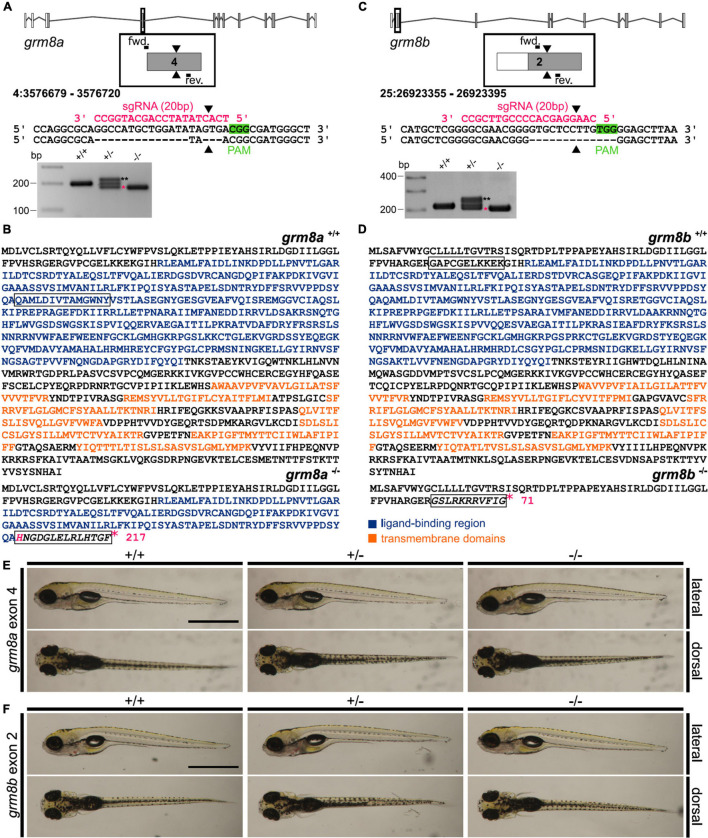
Generation of *grm8a* and *grm8b* knock-out lines using CRISPR/Cas9. **(A)** *Grm8a* exon-intron structure (top) with coding (gray) and non-coding exons (white). The sgRNA (pink) targeting *grm8a* exon 4 and the primer binding sites for genotyping PCR are displayed. The CRISPR/Cas9-induced strand break (black arrowheads) caused a 17 bp deletion represented by a smaller PCR product (pink asterisk) in *grm8a*^±^ and *grm8a^–/–^* animals (bottom). The third band of ∼220 bp corresponds to a heterodimer of wildtype and mutated PCR product (black asterisks). **(B)** The predicted amino acid sequence of the wildtype (top) and mutated allele (bottom) show that the frameshifted sequence (black box) is interrupted by a premature stop codon (pink asterisk) in the ligand binding domain. **(C)** *Grm8b* exon-intron structure (top) with coding (gray) and non-coding exons (white). The sgRNA (pink) targeting *grm8b* exon 2 and the primer binding sites for genotyping are indicated. The CRISPR/Cas9-induced strand break (black arrowheads) caused a 13 bp deletion represented by a smaller PCR product (pink asterisk) in *grm8b*^±^ and *grm8b^–/–^* animals (bottom). The third band of ∼250 bp corresponds to a heterodimer of wildtype and mutated PCR product (black asterisks). **(D)** The predicted amino acid sequence of the wildtype (top) and mutated allele (bottom) shows that the frameshifted sequence (black box) is interrupted by a premature stop codon (pink asterisk) in the N-terminal domain. **(E)** Dorsal (top) and ventral (bottom) views of 5 dpf old *grm8a^+/+^, grm8a^±^* and *grm8a^–/–^* zebrafish. Anterior is to the left. **(F)** Dorsal (top) and ventral (bottom) views of 5 dpf old *grm8b^+/+^, grm8b^±^* and *grm8b^–/–^* zebrafish. Scale bar, 1 mm.

Several CRISPR/Cas9 mutant lines were generated and one for each paralog was screened for morphological and behavioral phenotypes. The line for *grm8a*, which was chosen to be characterized further, contains a 17 bp deletion in *grm8a* exon 4 (Figure 5A and Supplementary Figure 4A). For *grm8b*, a line with a 13 bp deletion in exon 2 was selected (Figure 5C and Supplementary Figure 4D). In both cases, the CRISPR/Cas9-induced deletion mutation induces a frameshift and premature stop codon, leading to a termination of translation upstream (*grm8b*) or at the beginning of (*grm8a*) the ligand-binding domain in the very N-terminal region of the respective proteins potentially generating protein fragments of 217 and 71 aa, respectively (Figures 5B,D). The seven transmembrane and other functional domains are located more downstream of the target sites and are therefore missing in the truncated gene products (Figures 5B,D) making it unlikely that residual Grm8 activity is generated from the mutant alleles. We therefore consider these lines as potential null alleles.

We found that the gross morphology of *grm8a* (Figure 5E) and *grm8b* (Figure 5F) mutants is almost normal. Size measurements detect no significant alterations in head size, yolk diameter and body length of mutant embryos (Supplementary Figures 4B,E) except for a slight but not significant reduction in body length of *grm8b* mutants (Supplementary Figure 4E). Likewise, anti-cleaved Caspase 3 staining reveal no evidence for differences in cell apoptosis between mutants and wildtype siblings (Supplementary Figures 4C,F). In addition, there are no indications for early lethality in either of the mutant lines.

Next, we quantified the expression of both *grm8* transcripts in mutant animals. Whereas in *grm8a* mutants an allele-specific loss of *grm8a* transcript level was detected, indicating a probable non-sense-mediated decay, the *grm8b* transcript levels were unchanged, except for a slight, but not significant increase in *grm8a^–/–^* animals using the second primer pair located more downstream of the transcript in comparison to the first primer pair (Supplementary Figure 5A). However, in *grm8b^–/–^* animals a significant increase of *grm8b* expression using the second primer pair was detected, while *grm8a* transcript levels were unchanged (Supplementary Figure 5B). Due to the partial colocalization of *grm8a* with *gad1a* transcripts detected before (Figure 4), we decided to quantify expression levels of *gad1a*, *gad1b* and *gad2* in both mutant lines. In *grm8a^–/–^* animals a significant reduction of *gad1a* transcript level was detected, whereas *gad1b* and *gad2* were unchanged (Supplementary Figure 5A). In *grm8b* mutant animals no differences in *gad1a*, *gad1b* and *gad2* expression levels were found (Supplementary Figure 5B).

In conclusion, the overall wildtype-like appearance of *grm8a* and *grm8b* CRISPR/Cas9 mutants suggests that each of the Grm8 paralogs are not essential for cell survival, anatomical development, and survival in general.

### *Grm8a* and *Grm8b* Mutants Show Differential Locomotor and Thigmotaxis Phenotypes

**FIGURE 6:**
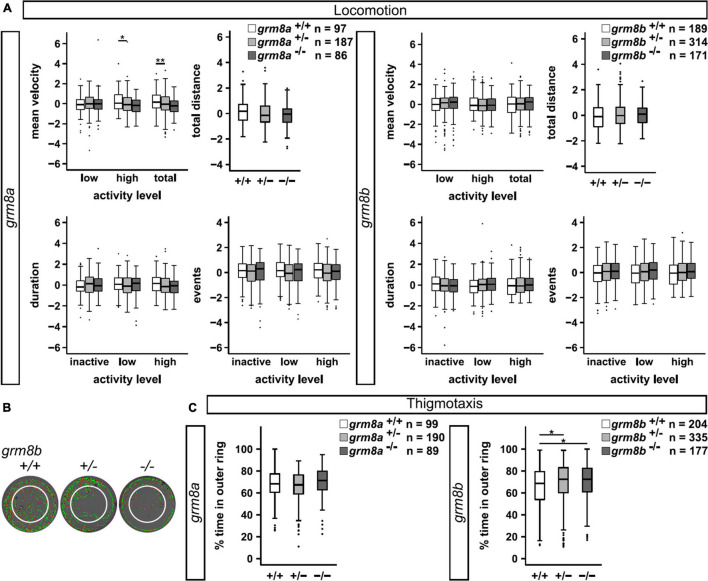
Behavioral characterization of *grm8a* and *grm8b* larvae. **(A)** Locomotor activity of *grm8a* (left panel) and *grm8b* mutant animals (right panel) were analyzed for mean velocity (top left) in low or high activity, or combined (total), total distance swum (top right), duration (bottom left) and events (bottom right) of inactivity, low and high activity. Raw data was standardized using z-score transformation. *P < 0.05, **P < 0.01. **(B)** Example traces of *grm8b^+/+^, grm8b^+/–^*, and *grm8b^–/–^* animals analyzed for thigmotaxis behavior. Note the reduced movement of mutant animals in the inner zone. Green represents slow, red fast movement. **(C)** Analysis of thigmotaxis behavior in *grm8a* (left panel) and *grm8b* (right panel) mutant animals. Time spent in the outer ring was analyzed for all genotypes and raw data standardized using z-score transformation. *P < 0.05.

In order to investigate the behavioral consequences of *grm8a* and *grm8b* loss-of-function we performed locomotor tracking at 5 dpf as described previously (Lüffe et al., 2021). Briefly, we placed larvae in a 12-well dish and recorded locomotor activity in a commercial setup for 5 min after 5 min of habituation. Activity levels were analyzed for the following parameters: mean velocity (low, high and total), total distance traveled, duration (inactive, low and high activity) and number of events counted for inactive, low and high activity for ^+/+^, ^+/–^ and ^–/–^ animals (Supplementary Table 3). For *grm8a*, significant differences in mean velocity were detected in mutant animals (Figure 6A), they exhibit a hypoactive phenotype characterized by a reduced “high” velocity and a general reduction in total mean velocity. A similar trend was observed for total distance swum and the duration and number of “high active” swimming events albeit without reaching statistical significance (Supplementary Table 3). In *grm8b* mutant animals, no differences in locomotor activity were detected (Figure 6B). Analysis of thigmotaxis behavior which is the tendency of animals to stay close to the walls of the wells and is usually considered to be associated with anxiety-like behavior were not altered for *grm8a* mutants compared to wildtype animals (Figure 6C). However, the *grm8b* mutants spend significantly more time in the outer ring compared to wildtypes suggesting an anxiety-like phenotype (Figures 6B,C). This initial characterization of *grm8a* and *grm8b* mutant behavior in zebrafish larvae points toward hypo-locomotion in *grm8a* mutants and an increase in fear-response in *grm8b* mutants, demonstrating that these mutant lines can be used to further characterize Grm8 function and elucidate its potential contribution to behavioral phenotypes with relevance for psychiatric disorders.

## Discussion

The current knowledge about Grm8 function in psychiatric disorders is scarce because the field lacks validated models in different species to address open questions. The present work provides such a model, describing the stepwise generation, validation and initial behavioral characterization of two paralog-specific mutant lines, on which future studies can now build. Further, with the presented colocalization of *grm8a* and *gad1a* expression, in particular in motor-related brain regions, the data highlights the GABAergic system as an interesting target for future investigations on the molecular link between *grm8a/grm8b* disruption and behavioral phenotypes implicated in neurodevelopmental and psychiatric conditions involving altered motor functions.

To study the role of Grm8 in neuronal circuits and its contribution to behavioral alterations in zebrafish, it is imperative to understand the spatial and temporal expression dynamics of both paralogs. We used whole-mount RNA ISH to identify spatial differences and similarities in the expression pattern of both genes. Since we are particularly interested in the potential role during neural development, we used early and late embryonic stages as well as adult brain tissue to monitor temporal changes in expression. Our data is in line with and complements the earlier study of Haug et al. (2013) in which embryonic expression was already demonstrated. Here, we provide a more detailed description to cover additional developmental stages and also include the expression pattern in the adult brain, which was not described before. Further, our research reveals a mutual expression in developing and adult brain regions involved in motor functions such as the subpallium, the posterior tuberculum, the thalamus, the cerebellum, and the medulla oblongata (with reticular formation, vagal and facial lobe). These results complement previous studies in zebrafish and other vertebrate species (Duvoisin et al., 1995; Saugstad et al., 1997; Shigemoto et al., 1997; Messenger et al., 2002; Haug et al., 2013; Ribeiro et al., 2017). Interestingly, we detected expression differences between *grm8a* and *grm8b* especially in the adult brain suggesting partial sub-functionalization of these two genes during evolution (Postlethwait et al., 2004). In contrast to *grm8b*, *grm8a* is strongly expressed in the reticular formation, the vagal nucleus, vagal and facial lobe. On the other hand, *grm8b* transcript is restricted to the facial and glossopharyngeal lobe, while a consistently stronger labeling of *grm8b* over *grm8a* can be exclusively found in the olfactory bulb. Thus, both *grm8a* and *grm8b* expression patterns will help to interpret phenotypic differences in *grm8a* and *grm8b* mutants in the future.

As the expression patterns for *grm8a* and *grm8b* partly resemble the distribution of GABAergic cells detected in previous studies (Lechermeier et al., 2019, 2020; Lüffe et al., 2021), we decided to use *gad1a* as a marker to prove the GABAergic identity of *grm8a* positive cells. We detected a partial colocalization of both transcripts at larval stages in brain regions associated with motor functions, including the subpallium, the posterior tuberculum, the thalamus, the cerebellum, and the medulla oblongata. There is evidence from the literature that indeed some GABAergic cells may express Grm8 protein. For instance, in mice and rat hippocampus it was shown that a subset of mGluR8a positive cells were of GABAergic origin and that presynaptic expression of mGluR8 in GABAergic boutons may modulate its inhibition depending on glutamatergic activity in this network (Ferraguti et al., 2005; Katona et al., 2020). Therefore, further functional experiments are needed to demonstrate that Grm8 is involved in the regulation of GABAergic activity also in zebrafish. In addition, our study has investigated the localization of *grm8a* and *gad1a* transcripts only. Subsequent research will have to include the paralogs as well.

In order to generate loss-of-function models we have chosen the guide RNA target sites to be located very early in the coding sequences of both genes. Even if translation would occur from the mutant transcripts, these fragments lack for instance the transmembrane regions so that both proteins would not reach their proper destination in the cell, would not get post translational modifications as needed and would not locate on the cell surface to act as a glutamate receptor. We therefore consider both lines to be functional null alleles. Due to the lack of proper antibodies recognizing zebrafish Grm8, we are not able to demonstrate the definite loss of protein expression in the mutant animals. Considering the sequencing results and expression differences detected with qPCR and the behavioral phenotypes we are confident that the mutants are indeed loss-of-function models.

The fact that both mutant lines are viable and do not display any gross morphological abnormalities or increased cell apoptosis indicates that, due to the similar expression pattern, the remaining *grm8*-gene might compensate for the loss of the other and therefore may prevent harsher phenotypes. This was partly reflected in our qPCR experiments where we observed a small, but not significant upregulation of *grm8b* transcript in *grm8a^–/–^* animals. Further, upregulation of *grm8b* transcripts was detected in *grm8b^–/–^* animals too, indicating a potential compensation for the loss of functional Grm8b protein in this mutant. Similar experiments with double mutant animals (*grm8a^–/–^; grm8b^–/–^*) in comparison to single mutants are needed to investigate this phenomenon further. In addition, other type III-mGluRs like mGluR4 or mGluR7 should be considered as potential compensators in future expression analyses due to the substantial similarities in their amino acid sequence and overlapping expression domains (Haug et al., 2013). The significant reduction of *gad1a* transcripts in *grm8a^–/–^* animals suggests that the GABAergic system might be affected by the Grm8a loss. The mechanistic background of this observation is currently unknown and needs to be addressed in future experiments.

To characterize the behavioral consequences of *grm8a* or *grm8b* loss, we performed an initial basic locomotor assay in the mutant lines. We detected a significant reduction in velocity in *grm8a* mutant larvae, indicating a hypoactivity phenotype. Hypoactivity was seen before in one mGluR8-knockout mouse model (Duvoisin et al., 2005), whereas other mouse models display no change in activity or hyperactivity phenotypes (Gerlai et al., 2002; Linden et al., 2002; Robbins et al., 2007; Raber and Duvoisin, 2015). The *grm8a* model could be useful in investigating motor related phenotypes further and resolving this conflicting data. Further unraveling the underlying mechanism(s) of this phenotype is of particular interest with respect to the observed *grm8a* and *gad1a* expression colocalization in brain regions involved in motor functions. The *grm8b* mutant animals did not show signs of motor activity alterations. Instead, they display a mild thigmotaxis phenotype. Thigmotaxis is considered to be an indicator for anxiety-like behavior, a phenotype which is not visible in *grm8a* mutants. Anxiety-like phenotypes were demonstrated for instance in a pharmacological model, in which mice were treated with the mGluR8-specific agonist (S)-3,4-dicarboxyphenylglycine. This induced decreased alcohol intake and produced an anxiolytic-like effect in the light-dark-box and open field assays. These effects were reversed with the type III-mGluR-specific antagonist (S)-2-amino-2-methyl-4- phosphonobutyric (Bahi, 2017), indicating that modulating mGluR8 activity with pharmacotherapy might be relevant in alcohol dependent disorders and anxiety. Also, the combination of mGluR8 with mGluR4 loss-of-function models could deliver stronger phenotypes and give deeper insights into disease mechanisms associated with type III-mGluRs (Raber and Duvoisin, 2015). In addition, it supports the proposed investigation of *grm4* transcript levels in *grm8a* and *grm8b* mutant lines within future research efforts. Altered fear responses are a frequent observation in mGluR8 knockout mice (Linden et al., 2002; Duvoisin et al., 2005; Robbins et al., 2007) and are correlated with the Grm8 deficiency in the amygdala (Schmid and Fendt, 2006; Fendt et al., 2013). Interestingly, *grm8a* and predominantly *grm8b* expression was detected in the zebrafish ventral/lateral pallium, which is assumed to comprise the homologous structure to the mammalian amygdala (Mueller et al., 2011; Ganz et al., 2014), opening the possibility that fear-related behavior in zebrafish larvae might be associated with *grm8a/grm8b* expression in the pallium. Therefore, our zebrafish *grm8b* model may be useful in elucidating the mechanism behind anxiety-like behavior associated with reduced Grm8 activity.

## Conclusion

Taken together, the two here newly developed animal models of Grm8 loss-of-function may be useful for the investigation of the mechanistic backgrounds of behavioral phenotypes with relevance to psychiatric disorders. Further research on double mutants and the characterization of adult animals are now the next logic steps. Several psychiatric disorders, including neurodevelopmental disorders, are linked to cellular processes that are thought to disturb the equilibrium of excitation and inhibition in the nervous system of patients, known as E/I imbalance (Canitano and Pallagrosi, 2017; Lopatina et al., 2019; Mamiya et al., 2021). This phenomenon might also be experimentally accessible in our loss-of-function lines, especially since we revealed expression of *grm8a* in GABAergic cell types. We anticipate that our two novel metabotropic glutamate receptor 8 zebrafish models may contribute to a deeper understanding of its function in development and disease.

## Data Availability Statement

The original contributions presented in this study are included in the article/Supplementary Material, further inquiries can be directed to the corresponding author.

## Ethics Statement

The animal study was reviewed and approved by the Regierung von Unterfranken (RUF 55.2.2 2532 2 622 10).

## Author Contributions

TL performed the experiments. ZG, MB, DÖ, CD, and CL contributed to experimental support. MR, CL, and CD contributed to conception and design of the study and supervised the project. TL and CD wrote the manuscript. All authors contributed to manuscript revision, read, and approved the submitted version.

## Conflict of Interest

The authors declare that the research was conducted in the absence of any commercial or financial relationships that could be construed as a potential conflict of interest.

## Publisher’s Note

All claims expressed in this article are solely those of the authors and do not necessarily represent those of their affiliated organizations, or those of the publisher, the editors and the reviewers. Any product that may be evaluated in this article, or claim that may be made by its manufacturer, is not guaranteed or endorsed by the publisher.

## Footnotes

1
http://cshprotocols.cshlp.org/content/2011/7/pdb.rec12467
