# Supplementary material for: Loss-of-Function Models of the Metabotropic Glutamate Receptor Genes Grm8a and Grm8b Display Distinct Behavioral Phenotypes in Zebrafish Larvae (Danio rerio)
Source: Front Mol Neurosci. 2022 Jun 13;15:901309. doi: 10.3389/fnmol.2022.901309 (PMC9234528; doi:10.3389/fnmol.2022.901309)
Supplement: Supplementary file 1 [file Data_Sheet_1.PDF]

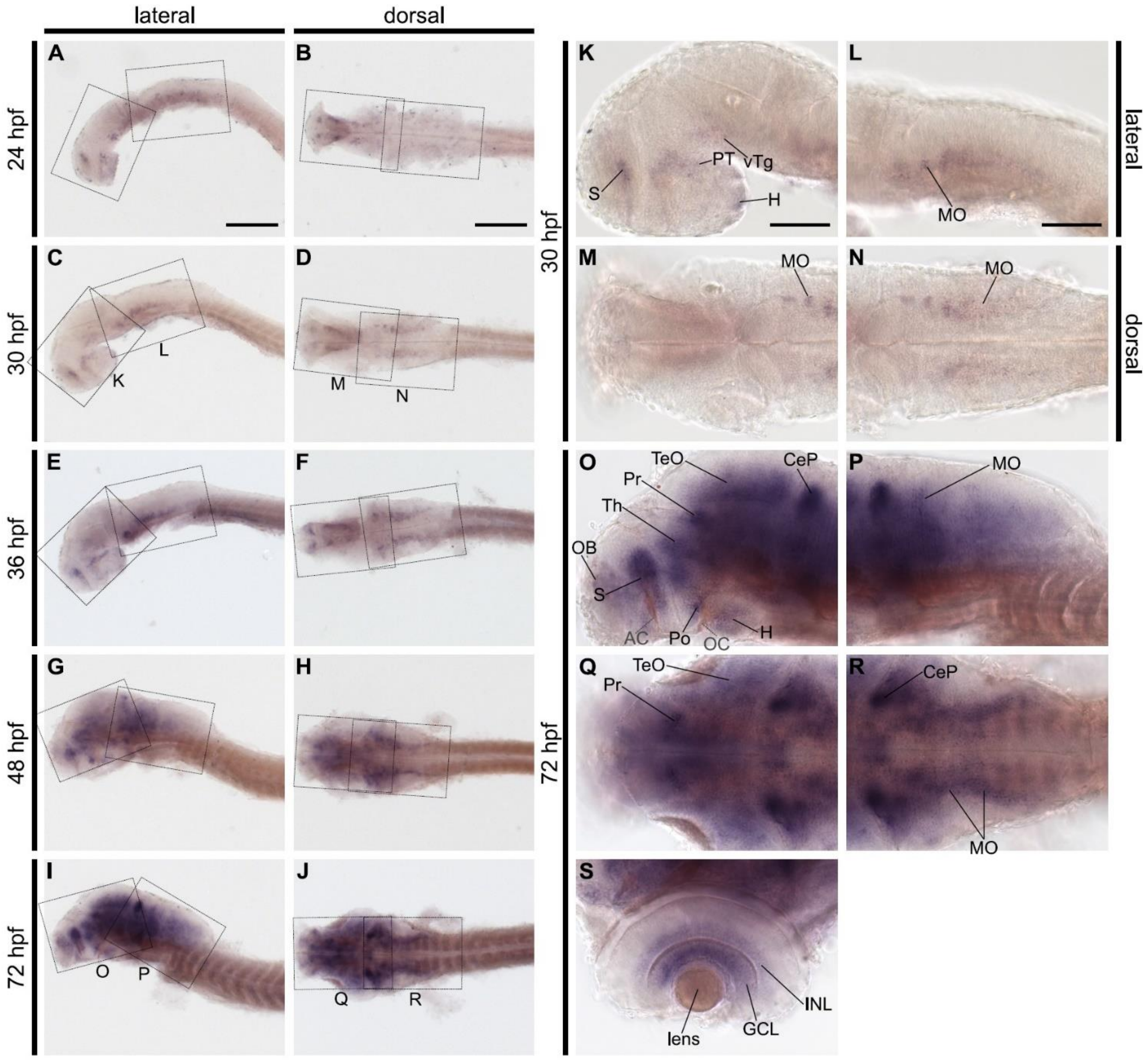

Supplementary Figure 1. *grm8a* expression pattern in the developing zebrafish revealed by whole-mount RNA *in situ* hybridization. Lateral (**A**, **C**, **E**, **G**, **I**) and dorsal (**B**, **D**, **F**, **H**, **J**) overview of 24, 30, 36, 48 and 72 hpf old wildtype zebrafish labelled for *grm8a* transcript (left panel). Boxed areas in C, D and I, J are magnified in K-R and displayed from lateral (**K**, **L**, **O**, **P**) and dorsal (**M**, **N**, **Q**, **R**) views (right panel). Remaining magnifications of boxed areas (24, 36 and 48 hpf) are shown in Figure 1. (**S**) *grm8a* transcript labelling in the eye from a dorsal view. All images are displayed with anterior to the left. Abbreviations are listed in Table 1. A detailed description of the developmental trajectory of *grm8a* expression in zebrafish is given in the main text. Scale bar, 200 µm (overview) and 100 µm (magnifications).

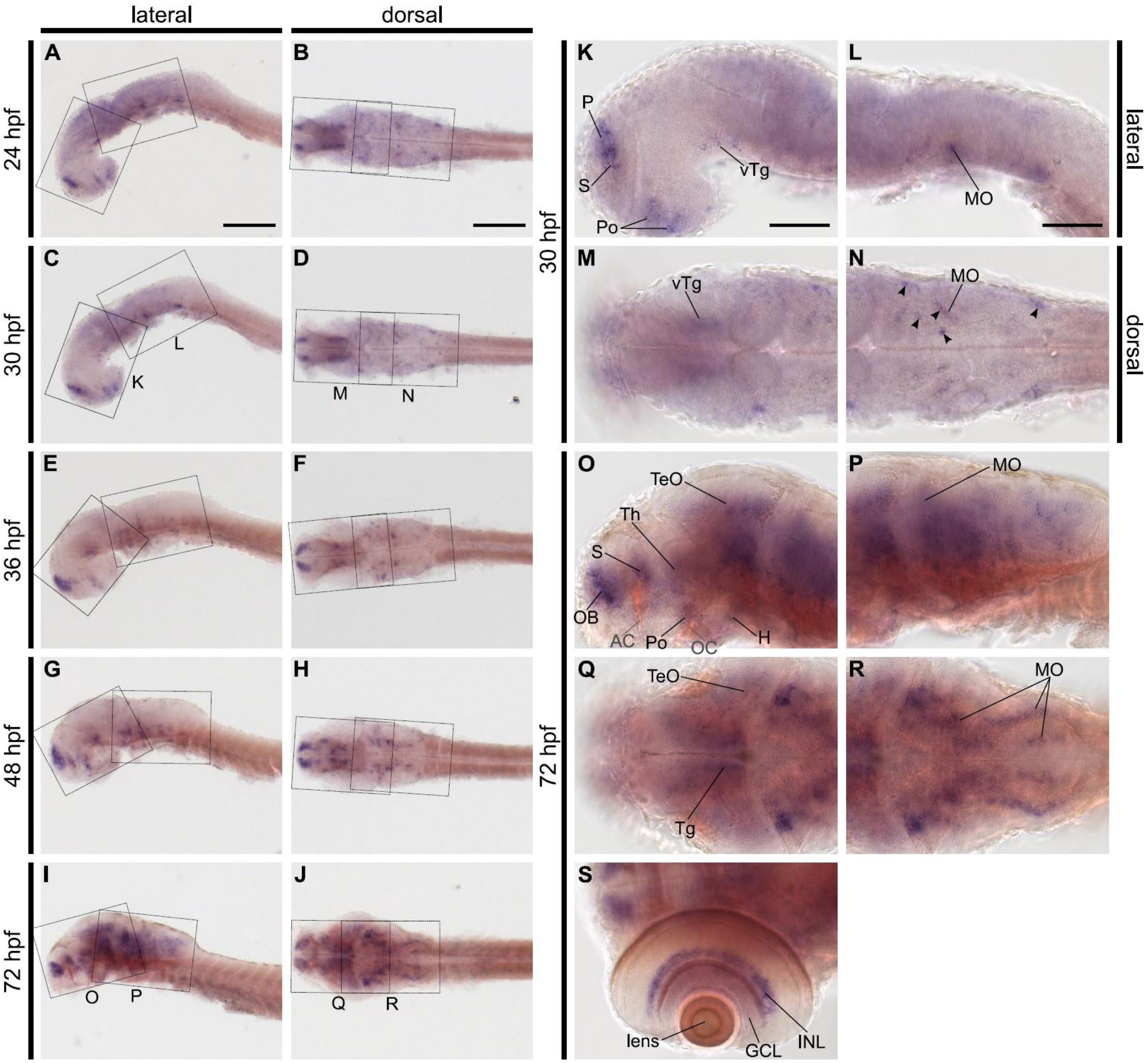

Supplementary Figure 2. *grm8b* expression pattern in the developing zebrafish revealed by whole-mount RNA *in situ* hybridization. Lateral (**A**, **C**, **E**, **G**, **I**) and dorsal (**B**, **D**, **F**, **H**, **J**) overview of 24, 30, 36, 48 and 72 hpf old wildtype zebrafish labelled for *grm8b* transcript (left panel). Boxed areas in C, D and I, J are magnified in K-R and displayed from lateral (**K**, **L**, **O**, **P**) and dorsal (**M**, **N**, **Q**, **R**) views (right panel). Remaining magnifications of boxed areas (24, 36 and 48 hpf) are shown in Figure 1. (**S**) *grm8b* transcript labelling in the eye from a dorsal view. All images are displayed with anterior to the left. Abbreviations are listed in Table 1. A detailed description of the developmental trajectory of *grm8b* expression in zebrafish is given in the main text. Scale bar, 200 µm (overview) and 100 µm (magnifications).

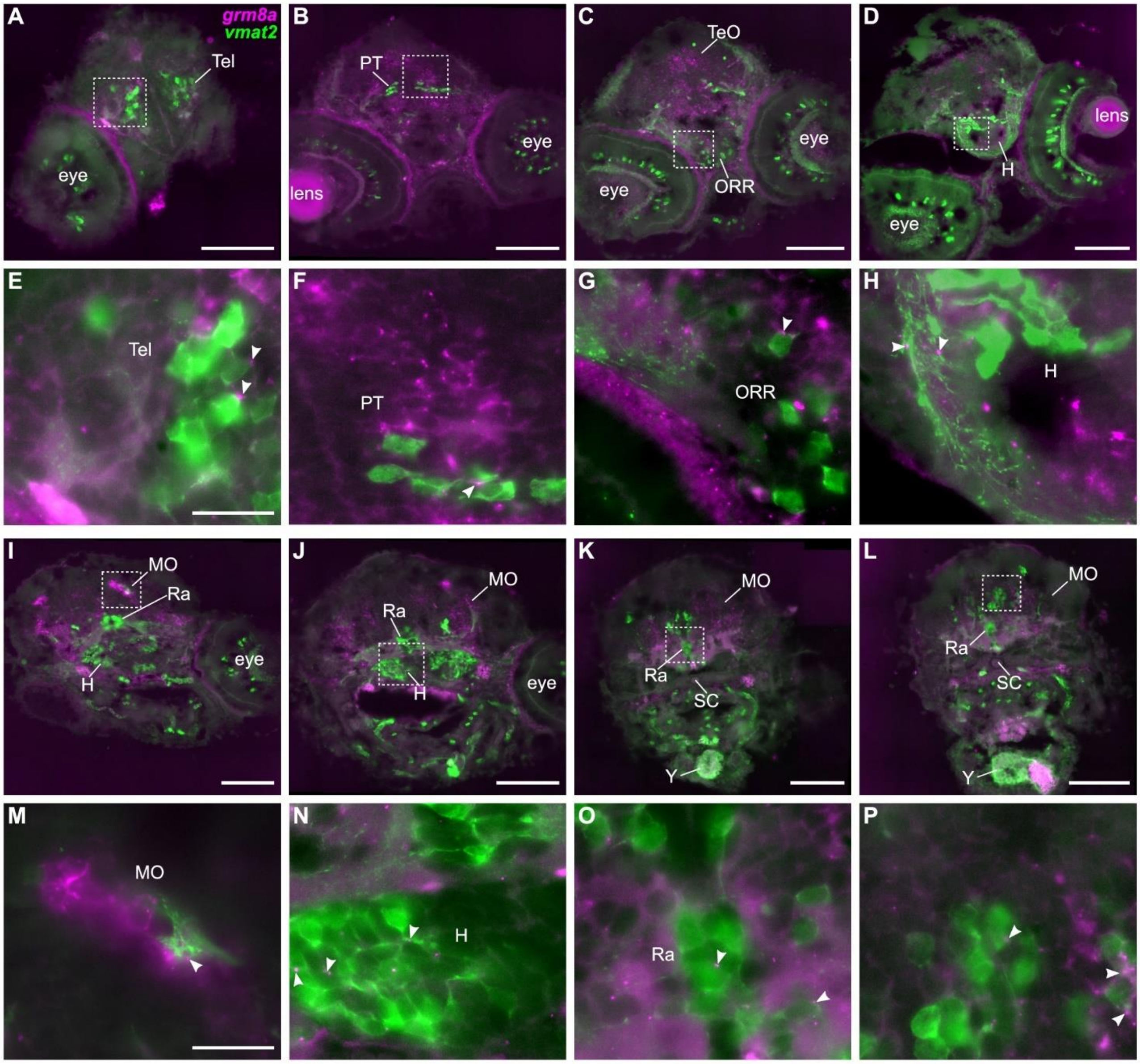

Supplementary Figure 3. Labeling of *grm8a* transcripts and *vmat2*-expressing cells in 72 hpf old transgenic zebrafish larvae. Tg(*Etvmat2:GFP*) larvae were labeled for *grm8a* transcripts by RNA in situ hybridization (shown in magenta) and expression of GFP (shown in green) by immunohistochemistry on cross-sections (**A-P**). Magnifications displayed in **E-H** and **M-P** represent the enlarged boxed areas in **A-D** and **I-L**. Clear colocalization or close proximity of *grm8a*-positive signals and vmat2:GFP expression are indicated by arrowheads. Anatomical abbreviations are listed in Table 1. Scale bar, 100 µm (overview) and 20 µm (magnifications). This figure was modified from the PhD thesis of Teresa Lüffe (Lüffe, 2021).

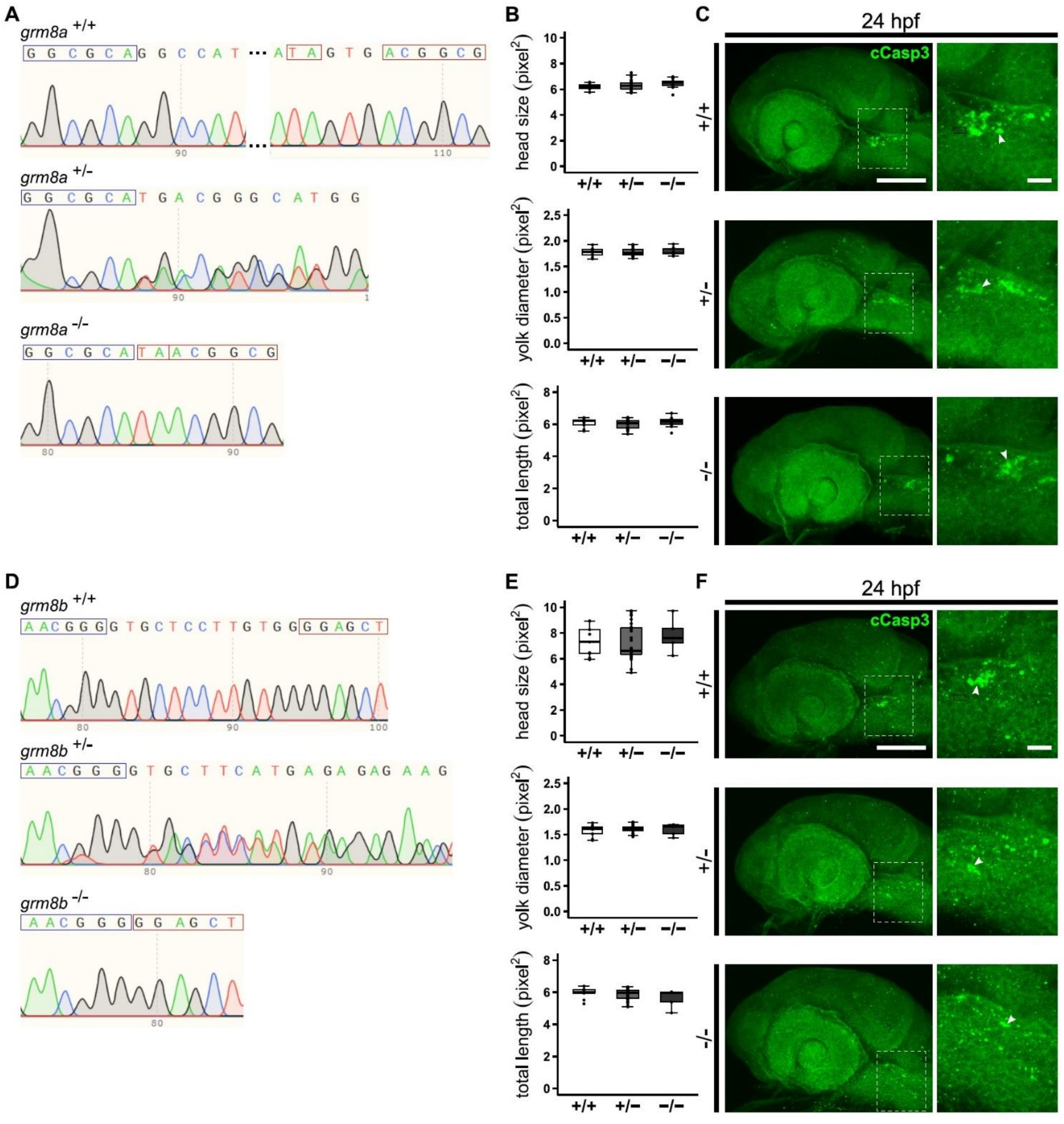

Supplementary Figure 4. Genetic and anatomical details of *grm8a* and *grm8b* mutants. (**A**) DNA sequencing traces for genotyping PCR product of *grm8a*$^{+/+}$, *grm8a*$^{+/-}$ and *grm8a*$^{-/-}$. *grm8a*$^{-/-}$ traces miss 17 nt in total confirming the deletion mutation described in Figure 5. *grm8a*$^{+/-}$ show two overlaying traces from the deletion mutation onwards representing a mixture of wildtype and mutated allele. (**B**) Measurements (in squared pixel (pixel^2)) of head area (top), yolk diameter (center) and total body length (bottom) in 24 hpf *grm8a*$^{+/+}$ (white, n=9), *grm8a*$^{+/-}$ (light grey, n=24) and *grm8a*$^{-/-}$ (dark grey, n=9). No significant size differences in head, yolk, or total length were observed between wildtype ($^{+/+}$) and heterozygous ($^{+/-}$) or homozygous ($^{-/-}$) *grm8a* mutant siblings. (**C**) No general increase in apoptosis in the central nervous system (CNS) of 24 hpf old *grm8a*$^{+/-}$ and *grm8a*$^{-/-}$

compared to *grm8a*$^{+/+}$ was revealed by anti-cleaved Caspase 3 staining (cCasp3). Magnifications of boxed areas in the left column are displayed to the right. Arrowheads indicate examples of cCasp3-positive (apoptotic) cells. (**D**) DNA sequencing traces for genotyping PCR product of *grm8b*$^{+/+}$, *grm8b*$^{+/-}$ and *grm8b*$^{-/-}$. *grm8b*$^{-/-}$ traces miss 13 nt in total confirming the deletion mutation described in Figure 5. *grm8a*$^{+/-}$ show two overlaying traces from the deletion mutation onwards representing a mixture of wildtype and mutated allele. (**E**) Measurements (in squared pixel (pixel^2)) of head area (top), yolk diameter (center) and total body length (bottom) in 24 hpf *grm8b*$^{+/+}$ (white, n=9), *grm8b*$^{+/-}$ (light grey, n=29) and *grm8b*$^{-/-}$ (dark grey, n=5). No significant size differences in head, yolk, or total length were observed between wildtype ($^{+/+}$) and heterozygous ($^{+/-}$) or homozygous ($^{-/-}$) *grm8b* mutant siblings. (**F**) No general increase in apoptosis in the central nervous system (CNS) of 24 hpf old *grm8b*$^{+/-}$ and *grm8b*$^{-/-}$ compared to *grm8b*$^{+/+}$ was revealed by anti-cleaved Caspase 3 staining (cCasp3). Magnifications of boxed areas in the left column are displayed to the right. Arrowheads indicate examples of cCasp3-positive (apoptotic) cells. Images are oriented with anterior to the left. Scale bar, 100 µm. This figure was modified from the PhD thesis of Teresa Lüffe (Lüffe 2021).

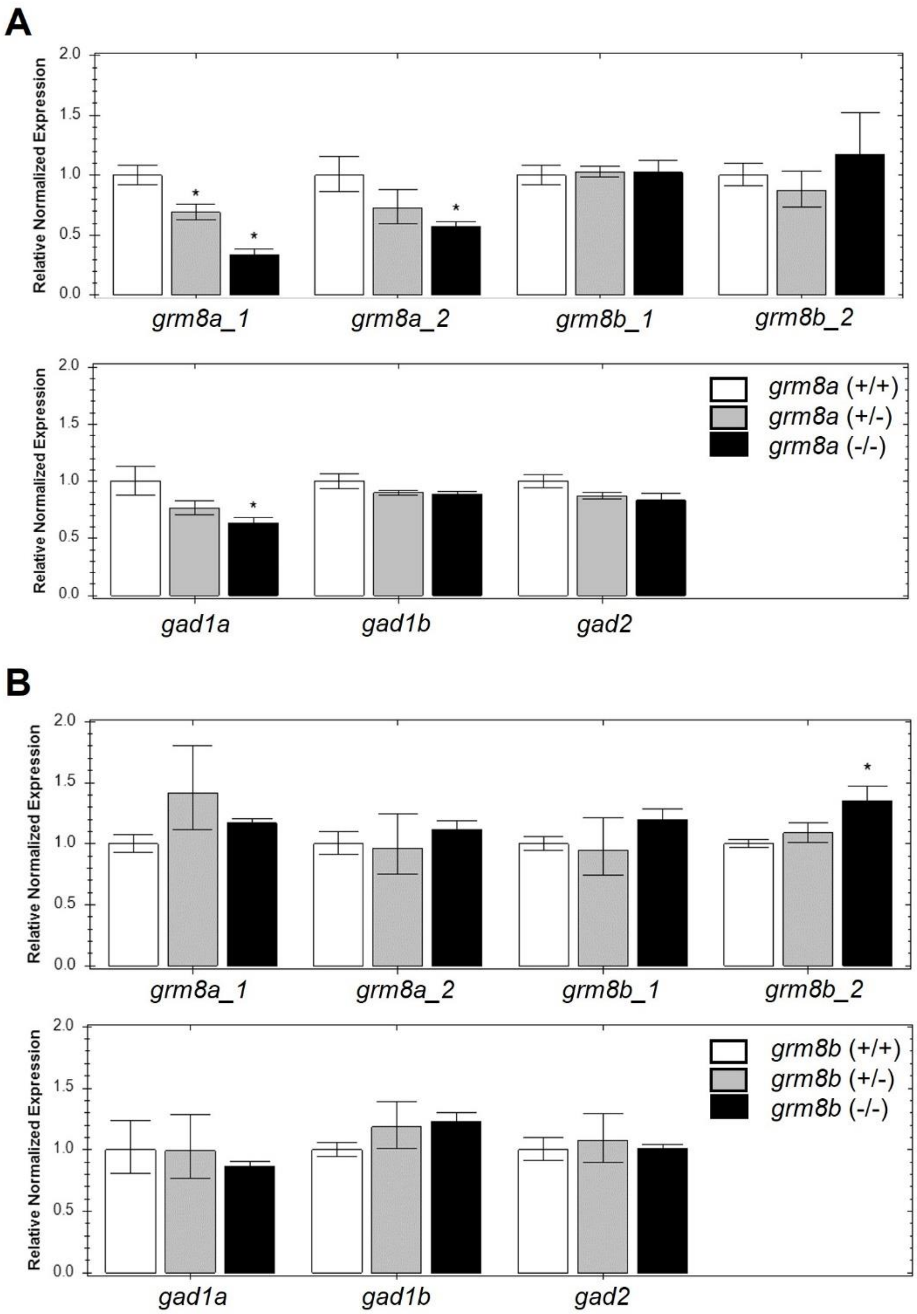

Supplementary Figure 5. Relative normalized expression of *grm8a*, *grm8b*, *gad1a*, *gad1b* and *gad2* in *grm8a* and *grm8b* mutant animals and wildtype siblings. **(A)** qPCR analysis in *grm8a* mutant animals display allele-specific decrease of *grm8a* transcripts, indicating nonsense-mediated decay (upper panel). In *grm8a*$^{-/-}$ animals *gad1a* expression was significantly reduced (lower panel). **(B)** qPCR analysis in *grm8b* mutant animals showed induction of expression of *grm8b* with the grm8b_2 primer pair (upper panel). No changes in expression were detected in the expression of the *gad1a*, *gad1b* and *gad2* transcripts (lower panel). All amplicons were intron-spanning. Two independent primer pairs were used for *grm8a* and *grm8b* transcripts, covering distinct parts and splice sites in the transcript. *P < 0.05.

Oligonucleotides	Sequences (5'-3')	Amplicon size (bp)	Annealing temperature (°C)
**Cloning sgRNAs**			
GR_grm8a_ex4_a<br>GR_grm8a_ex4_b	-TAGGCCATGCTGGATATAGTGA-<br>-AAACTCACTATATCCAGCATGGCC-	-	-
GR_grm8b_ex2_a<br>GR_grm8b_ex2_b	-TAGGCGAACGGGGTGCTCCTTG-<br>-AAACCAAGGAGCACCCCGTTCGCC-	-	-
**Realtime qPCR**			
RT_gapdh_a<br>RT_gapdh_b	-CGATCACTTTGTCAAGCTGGT-<br>-GCTGTAACCGAACTCATTGTCA-	52	61
RT_actb1_a<br>RT_actb1_b	-GCTGTTTTCCCCTCCATTGTT-<br>-GGGCCTCATCTCCCACATAG-	91	61
RT_grm8a_1a<br>RT_grm8a_1b	-AGGAAGCCTGAAAAAGTGCAC-<br>-ATAACTAGAGTCCCGGCCCA-	55	61
RT_grm8a_2a<br>RT_grm8a_2b	-GGGAAAGAGCTGCTGGGATA-<br>-AAGACCACTGGAGTTCCTGC-	68	61
RT_grm8b_1a<br>RT_grm8b_1b	-TCTGCGAGAGTGTGGACAAT-<br>-AGGATGTTCGCTCATATGGCA-	85	61
RT_grm8b_2a<br>RT_grm8b_2b	-GGCCAGTGAACGACCCAAT-<br>-TGGCATGATTGGTGTAGCTGA-	107	61
RT_gad1a_a<br>RT_gad1a_b	-CAGATGGAGAGGAGAAACGACAT-<br>-ACCATTGTTGTCCCGCACT-	85	61
RT_gad1b_a<br>RT_gad1b_b	-ATGCCGAACGGAGACGAG-<br>-GCACTCCATCATCATTGCTTTG-	75	61
RT_gad2_a<br>RT_gad2_b	-GTGGAGAGGATGAAGCGTCTG-<br>-GACCATGCGGAAGAAGTTGAC-	123	61
**Genotyping**			
GT_grm8a_in3a<br>GT_grm8a_ex4b	-CTTCTTTCGTCTGCAGATTCCT-<br>-TTTCCCCATAGTTTCCTTCAGA-	194 (wt),<br>177 (KO)	55
GT_grm8b_ex2a<br>GT_grm8b_ex2b	-GTACGGATCCACTGACTCCC-<br>-GTAACATTGGGCAGCAGGTC-	217 (wt),<br>204 (KO)	58

Supplementary Table 1. Oligonucleotides used for cloning of sgRNA constructs, qPCR and genotyping of mutant animals.

	OB	Tel	S	P	Po	Th	Pr	H	PT	vTg	TeO	CeP	MO	GCL	INL
grm8a															
24 hpf		x								x					
30 hpf			x					x	x	x			x		
36 hpf			x		x	x		x	x	x			x		
48 hpf			x		x	x		x	x	x		x	x		
72 hpf	x		x		x	x	x	x	x	x	x	x	x	x	x
overlap *gad1a*															
overlap *vmat2:GFP*															
grm8b															
24 hpf		x						x		x			x		
30 hpf			x	x	x			x		x			x		
36 hpf	x		x	x	x			x		x			x		
48 hpf	x		x	x	x			x		x			x		
72 hpf	x		x		x	x		x		x	x		x	x	x

Supplementary Table 2. Summary of the expression patterns of *grm8a* and *grm8b* transcripts for 5 different developmental stages and its regional distribution in embryonic and larval brain regions. The overlap of *gad1a* expression is shown in orange (at 36 hpf, 48 hpf, and/or 72 hpf) and regional overlap with *vmat2:GFP* (at 72 hpf) is marked in yellow in *grm8a* labeled animals. Temporal differences of (possibly) low relevance due to differences in staining quality are marked in grey. Temporal differences between both expression patterns are highlighted in blue. Distinct expression domains are indicated in red. Anatomical abbreviations are listed in Table 1. This table was modified from the PhD thesis of Teresa Lüffe (Lüffe, 2021).

grm8a	**+/+ vs. +/-**	**+/+ vs. -/-**	**+/- vs. -/-**
total distance	0.08	0.14	0.80
mean velocity (low)	0.98	0.75	1.00
mean velocity (high)	**0.04**	0.16	0.23
mean velocity (total)	**0.01**	0.13	0.11
duration (inactive)	0.73	0.22	0.74
duration (low)	0.87	0.41	0.31
duration (high)	0.10	0.19	0.78
events (inactive)	0.64	0.48	0.41
events (low)	0.65	0.43	0.56
events (high)	0.38	0.26	0.73
% time in outer ring	0.70	0.21	0.07
grm8b	**+/+ vs. +/-**	**+/+ vs. -/-**	**+/- vs. -/-**
total distance	0.42	0.38	0.59
mean velocity (low)	0.14	0.23	0.87
mean velocity (high)	0.94	1.00	0.77
mean velocity (total)	1.00	0.83	0.85
duration (inactive)	0.28	0.25	0.60
duration (low)	0.09	0.18	0.80
duration (high)	0.73	0.72	0.54
events (inactive)	0.29	0.24	0.63
events (low)	0.05	0.21	0.52
events (high)	0.22	0.30	0.45
% time in outer ring	**0.04**	**0.04**	0.89

Supplementary Table 3. Corresponding p-values for behavioral results. Statistically significant differences based on p-values are labelled in bold.

grm8a	**+/+ vs. +/-**	**+/+ vs. -/-**	**+/- vs. -/-**
mean velocity (high)	**0.11 [0.03-0.25]**	0.21 [0.04-0.36]	n/a
mean velocity (total)	**0.11 [0.03-0.24]**	0.25 [0.08-0.4]	n/a
grm8b	**+/+ vs. +/-**	**+/+ vs. -/-**	**+/- vs. -/-**
% time in outer ring	**0.10 [0.00-0.20]**	**0.12 [0.00-0.23]**	n/a

Supplementary Table 4. Effect size given as Cohen's d. Effect sizes for statistically significant differences (based on previously calculated p-values) are labelled in bold.
